# Chronic stress targets mitochondrial respiratory efficiency in the skeletal muscle of C57BL/6 mice

**DOI:** 10.1007/s00018-023-04761-4

**Published:** 2023-03-29

**Authors:** Aleksandra Nikolic, Pia Fahlbusch, Natalie Wahlers, Nele-Kathrien Riffelmann, Sylvia Jacob, Sonja Hartwig, Ulrike Kettel, Matthias Dille, Hadi Al-Hasani, Jörg Kotzka, Birgit Knebel

**Affiliations:** 1 Institute of Clinical Biochemistry and Pathobiochemistry, German Diabetes Center at the Heinrich-Heine-University Duesseldorf, Leibniz Center for Diabetes Research, 40225 Duesseldorf, Germany; 2 German Center for Diabetes Research (DZD), Partner Duesseldorf, 40225 Duesseldorf, Germany; 3 Medical Faculty Heinrich-Heine-University Düsseldorf, 40225 Düsseldorf, Germany

**Keywords:** Metabolic adaptation, Proteome, Transcriptome, Methylome, Enriched mitochondria, Mitochondrial thermodynamic efficiency (ƞ-opt)

## Abstract

Episodes of chronic stress can result in psychic disorders like post-traumatic stress disorder, but also promote the development of metabolic syndrome and type 2 diabetes. We hypothesize that muscle, as main regulator of whole-body energy expenditure, is a central target of acute and adaptive molecular effects of stress in this context. Here, we investigate the immediate effect of a stress period on energy metabolism in *Musculus gastrocnemius* in our established C57BL/6 chronic variable stress (Cvs) mouse model. Cvs decreased lean body mass despite increased energy intake, reduced circadian energy expenditure (EE), and substrate utilization. Cvs altered the proteome of metabolic components but not of the oxidative phosphorylation system (OXPHOS), or other mitochondrial structural components. Functionally, Cvs impaired the electron transport chain (ETC) capacity of complex I and complex II, and reduces respiratory capacity of the ETC from complex I to ATP synthase. Complex I-OXPHOS correlated to diurnal EE and complex II-maximal uncoupled respiration correlated to diurnal and reduced nocturnal EE. Bioenergetics assessment revealed higher optimal thermodynamic efficiencies (ƞ-opt) of mitochondria via complex II after Cvs. Interestingly, transcriptome and methylome were unaffected by Cvs, thus excluding major contributions to supposed metabolic adaptation processes. In summary, the preclinical Cvs model shows that metabolic pressure by Cvs is initially compensated by adaptation of mitochondria function associated with high thermodynamic efficiency and decreased EE to manage the energy balance. This counter-regulation of mitochondrial complex II may be the driving force to longitudinal metabolic changes of muscle physiological adaptation as the basis of stress memory.

**Supplementary Information:**

The online version contains supplementary material available at 10.1007/s00018-023-04761-4.

## Introduction

Epidemiological studies show that chronically elevated levels of stress hormones are a risk factor for diabetes, obesity, insulin resistance, and metabolic syndrome [1–5]. Threatening life situations and chronic stress can result in posttraumatic stress syndromes, which is not only a psychic condition, but a predisposition to metabolic disease and thus have a general clinical implication for the pathogenesis of obesity, insulin resistance, or type 2 diabetes to the development and progression of fatty liver [1–5]. Since glucocorticoid (GC) measurements can be used as a stress indicator and GC also contributes to metabolic regulation by promoting hepatic gluconeogenesis, decreasing insulin sensitivity, and inducing adipose tissue lipolysis [1, 3, 5].

However, the stress response is not synonymous with GC response but is a bundle of coordinated factors and processes that act in an energy-dependent manner to adapt to stress. Stress processes, therefore, interfere with energy homeostasis in energy provision, energy consumption, or substrate utilization, bringing the focus to mitochondria. This is further supported by the fact that all components of stress regulation are synthesized and regulated in mitochondria via key pathways [6].

Over 90% of cellular energy production occurs in the mitochondria via oxidative phosphorylation [7], but several essential metabolic pathways, such as ß-oxidation of fatty acids, tricarboxylic acid (TCA) cycle, stress and sex hormone synthesis, as well as urea cycles, are also located in this organelle. In addition, environmental factors, such as stress, can affect mitochondrial activity, and mitochondria themselves act as signal transducing organelles through intermediates derived from the TCA cycle, metabolites, nucleotides or reactive oxygen species (ROS) [8]. These metabolic intermediates affect various cellular metabolic processes and also epigenetic DNA modifications, creating a direct link from mitochondrial function to transcriptional regulation and epigenetic processes [9–12]. Given their crucial role in cell physiology, it is evident that mitochondria have become the first responders to various stressors that affect the homeostasis of the cell and the organism. At least, mitochondrial function is involved in the adaptation of cellular metabolism and the development and progression of metabolic diseases such as obesity, insulin resistance, and type 2 diabetes [13–15].

The skeletal muscle is a key organ of whole body glucose homeostasis and whole-body energy expenditure (EE) is coupled to the capacity of mitochondrial respiration in skeletal muscle [16, 17]. When mitochondria are under pressure to adapt metabolism in response to exacerbating health-threatening situations, there is a gradual decline in mitochondrial organelle function. This can range from changes in electron transport chain (ETC) activity and potential, to functional impairments, like proton leakage, decrease in ATP production, increasing membrane potential, mitophagy, mitochondrial and genomic gene expression, up to changes in cellular metabolic processes and even programmed cell death [18–21]. Additional or sustained high-energy demands can lead to increased organelle activity and further to compensatory increased mitochondrial biogenesis, mitochondrial DNA content and damage, and eventual organelle decline [18, 22]. Moreover, insufficient oxidative capacity due to environmental stressors e.g. insulin resistance can induce mitochondrial stress leading to mitochondrial dysfunction [23–25].

To date, the mechanisms involved in the dysregulation of energy metabolism and mitochondrial function following chronic stress suggested as basis for the metabolic late effects are still under debate [2, 5, 26]. Chronic variable stress (Cvs) affects whole-body energy metabolism but it is tempting to speculate that it acts in a tissue-specific manner on a molecular level.

We have previously shown that chronic stress causes hepatic insulin resistance but longitudinal development of improved insulin sensitivity in peripheral tissues [26]. Therefore, it came clear that there have to be tissue-specific mechanisms resulting in long-term metabolic effects of Cvs. Immediately after Cvs, glucose- or lipid uptake and insulin signaling were unaltered in muscle tissues in our initial study [26]. However, measurements of palmitate oxidation, β-oxidation-linked, as well as TCA-cycle-linked respiration show longitudinal effects in muscle tissue, while mitochondrial mass remains unaltered [26]. The data show an overall tendency towards increased energy metabolism in muscle tissue concerning different substrate utilization pathways, which may indicate a general reorganization of mitochondrial metabolic pathways. Here, we follow this observation in detail to account for the molecular mechanisms initiated in muscle directly after Cvs that induce the ongoing molecular adaptation to metabolic stress processes.

In the present study, we examined the immediate effects of Cvs intervention on skeletal muscle, specifically *M. gastrocnemius*. Changes in physical activity, energy expenditure, transcriptome, methylome, proteome, and extracellular flux analysis to determine oxygen consumption rate in isolated skeletal muscle mitochondria, detailed mitochondrial complex capacity and mitochondrial thermodynamics were used to evaluate stress-mediated effects. Our results suggest that mitochondrial disturbance right after Cvs is accompanied by reduced EE and is compensated by an increase in complex II thermodynamic coupling and its efficiency of oxidative phosphorylation. This may indicate an early stage of mitochondrial adaptation to Cvs as a compensatory mechanism to manage the energy balance.

## Materials and methods

### Experimental animals

C57BL/6 male mice (12 weeks old) were housed (5–6 animals per cage) within standard laboratory conditions (12 h light/12 h night cycles, 22–24 °C, with ad libitum access to tap water and standard chow food (R/M-H extrudate: 58% carbohydrates, 9% fat, 33% protein; ssniff Spezialdiäten GmbH, Soest, Germany). All procedures were approved (LANUV, NRW, Germany (81–02.04.2017.A421)) and carried out in accordance with the ‘Principle of laboratory animal care’ (NIH publication No. 85–23, revised 1996) and the German law on the protection of animals. One group of C57BL/6 animals was exposed to our established chronic variable stress (Cvs) protocol [26, 27]. In brief, stressors were: (i) individual caging on a shaker (100 rpm, 1 h); (ii) 30 min restrain; (iii) individual caging without bedding (4 °C, 1 h); (iv) swimming in warm water (30 °C, 20 min); and (v) overnight housing in a large cage. The control group was untreated and only subjected to gentle handling during the weaning and ranking phase until 15 weeks of age. After the mice were sacrificed by CO_2_ asphyxiation, blood was collected by cardiac puncture, and muscle biopsies (*M. gastrocnemius*) were immediately removed for isolation of mitochondrial fractions. Muscle tissue for methylome and RNA analyses was snap-frozen in liquid nitrogen and stored at − 80 °C until further processing.

### Body composition

Body composition was analyzed (*n* = 6 animals per group) using nuclear magnetic resonance (NMR) (Whole Body Composition Analyzer, Echo MRI, TX, USA) to determine fat and lean mass before and after 15 days of the Cvs intervention. Body composition was measured by a sequence of radio pulses followed by nuclear magnetic resonance (NMR) echo recording. The sequence contains several periodic Carr-Purcell-Meiboom-Gill (CPMG) segments separated by pauses of varying duration. The characteristic (relaxation) time scales of the NMR responses (transverse "T2" and longitudinal "T1" relaxation) specific for fat mass and lean mass were analyzed. The final values of these parameters are calculated by linear regression based on the linear combination of the fat mass and lean mass and their respective relaxation rates (n = 6 animals per group) [28].

### Indirect calorimetry

Animals (*n* = 6 animals per group) were assessed for activity, respiratory exchange ratio (RER), and energy expenditure (EE) in metabolic cages (PhenoMaster, TSE Systems, Bad Homburg, Germany). To ensure acclimatization, the animals were placed in the cages 24 h before the start of the measurement recordings. Measurements began at 06:00 am and were taken every 30 min over a 72 h period. Analyses were calculated from data of two complete circadian cycles (48 h) starting with the first light phase. The mean values of 48 h were used. Measurements were performed with a non-invasive infra-red light beam system and the parameters analyzed were real-time feeding and physical activity. Animals had free access to food and water at 22 °C. Total energy expenditure (EE) was derived from oxygen consumption (VO_2_) and normalized by body surface area [29]. Respiratory exchange ratio (RER) was calculated from oxygen consumption (VO_2_) and carbon dioxide (VCO_2_) production. Furthermore, the carbohydrate oxidation (CHO) and fatty acid oxidation (FAO) rates were calculated according to Perronnet and Massicote [30]. The measurements were performed according to the manufacturer's recommendation.

### Plasma analyses

Plasma was immediately obtained by centrifugation from collected blood (2000 xg, 10 min, 4 °C). Efficiency of our Cvs protocol was monitored by increased plasma corticosterone levels determined by radioimmunoassay (Corticosterone Double Antibody 125I RIA kit; MP Biomedicals, Orangeburg, NY, USA), increased fasting blood glucose (Contour, Bayer AG, Leverkusen, Germany) after a 6-h fasting as well as increased triglyceride and NEFA levels (Randox Triglycerides (RANDOX Laboratories, Antrim, United Kingdom) and Autokit NEFA C (Wako, Neuss, Germany)) (Supplement Fig. 1). All procedures were performed according to the manufacturer’s protocols. Plasma analyses were analyzed as means ± 95% CI of 6 animals per group. A Shapiro–Wilk analysis confirmed that the sample data were normally distributed, so data were analyses using parametric unpaired t-test.

**Fig. 1:**
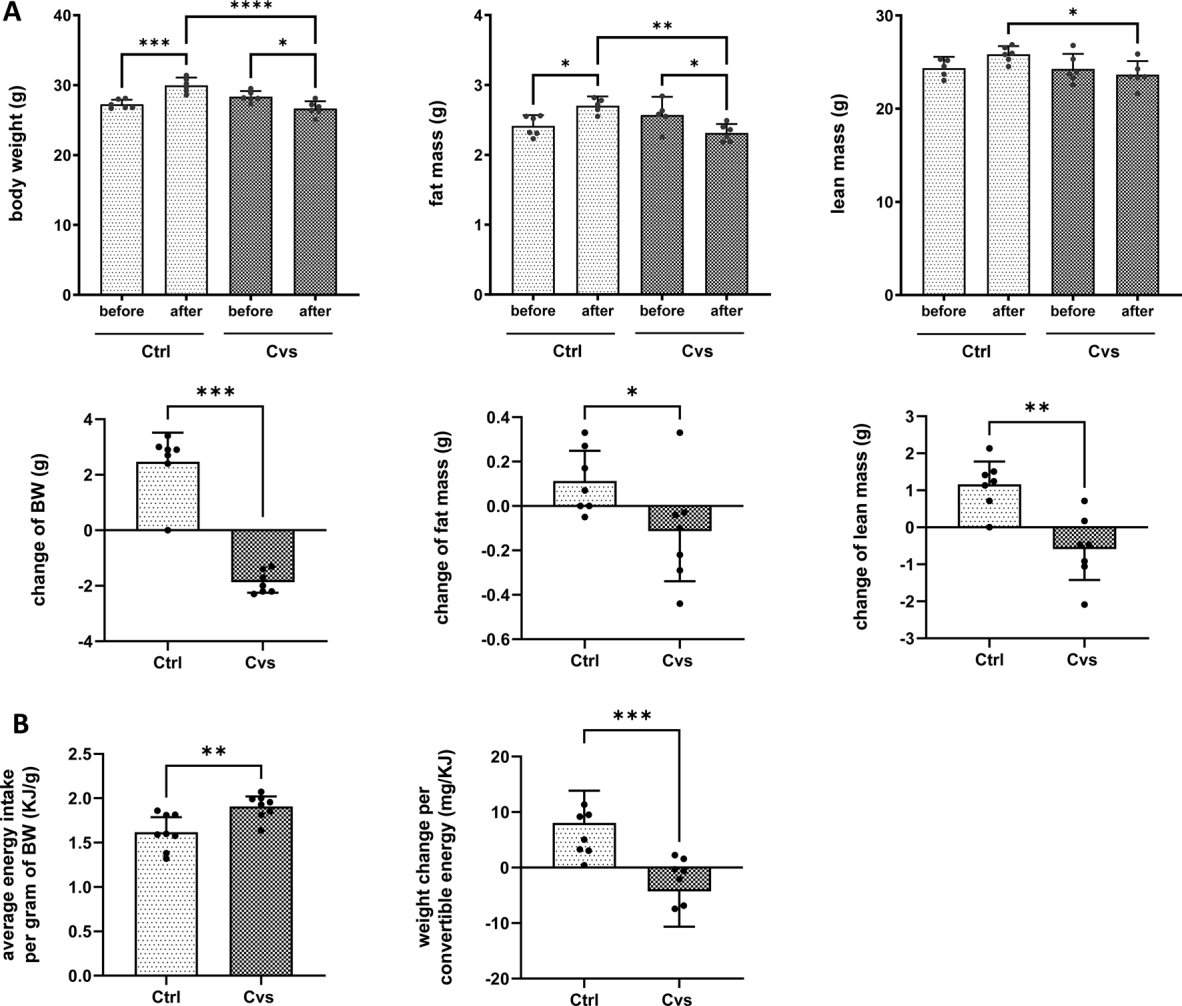
Effect of 15 days of chronic stress intervention (Cvs) on weight change, body composition, and food uptake. **A** NMR-determined analysis of body composition, consisting of body weight (BW), lean mass, and fat mass, before and after chronic variable stress in control (Ctrl) and Cvs mice (*n* = 6/group). **B** Differences in mean energy intake per gram of body weight (BW) and weight change per convertible energy are presented as mean of 8 measurements taken during the intervention period per group (*n* = 6/group). For all analyses of physiological changes, bar graphs represent the mean ± 95% CI. Single measurements of each animal or group are shown as dots. Statistics: one-way ANOVA with Tukey test for multiple comparisons or Mann–Whitney test, *p < 0.05, **p < 0.01, ***p < 0.001, ****p < 0.0001, Ctrl: C57BL/6, Cvs: C57BL/6 after stress intervention

### Enrichment of mitochondria

Enrichment of mitochondria [31] was modified for muscle tissue according to [32]. In brief, isolation was based on the differential centrifugation method by 100 mg of *M. gastrocnemius* minced at a ratio of 1:5 (w/v) in a hypoosmotic buffer (100 mM KCl, 50 mM MOPS, 1 mM EDTA, 5 mM MgSO_4_, 0.5% fatty-acid free BSA, pH = 7.4) using a homogenizer (TissueRuptorII, Qiagen, Hilden, Germany). The lysate was cleared (600×*g*, 20 min, 4 °C; (referred as 600×*g* fraction)) and the resultant supernatant was centrifuged at 10,000×*g* (20 min, 4 °C) to collect the enriched mitochondria. The pellet was resuspended in isolation buffer without BSA (100 mM KCl, 50 mM MOPS, 1 mM EDTA, 5 mM MgSO_4_, pH = 7.4) (referred as 10,000×*g* fraction). Protein concentration was determined using the BCA method.

### Analyses of mitochondria respiration and enzyme activity

Mitochondrial respiration experiments were performed by extracellular flux analysis using the Seahorse XFe24 instrument (Agilent, Waldbronn, Germany) as described [31]. 2.5 µg freshly enriched mitochondrial 10,000 xg fraction were added to each well. For electron flow measurement the substrate concentrations were 10 mmol/l pyruvate, 5 mmol/l malate, 10 mmol/l succinate, and 6 µmol/l FCCP. The initial inhibition of complex I was achieved by injection of 2 µmol/l rotenone and the inhibition of complex II was achieved by 10 mmol/l malonate. Complex III was inhibited by the injection of 2 µmol/l antimycin A, followed by the stimulation of complex IV by 100 µmol/l TMPD (*N*, *N*, *N*′, *N*′-Tetramethyl-1,4-phenylenediamine) in combination with 10 mmol/l ascorbate. The coupling assay was performed in presence of complex I- or complex II-specific substrates pyruvate/malate or succinate in the assay medium. The basal respiration (state 2) was mediated by complex I and complex II substrates. The oxidative phosphorylation (state 3) was initiated by 2 mmol/l ADP injection, the resting respiration rate (state 4o) was initiated by 2 µmol/l oligomycin injection and state 3u was mediated by 6 µmol/l FCCP injection. 2 µmol antimycin A injection induced complex III inhibition (all compounds purchased from Sigma Aldrich/MERCK, Germany). Enriched mitochondria fractions were allowed to equilibrate in the assay setup with two repetitive cycles, where one cycle corresponds to 30 s mix of substrate in the assay wells and 3 min rest of mitochondria in the transient microchamber. Two initial basal oxygen consumption rate (OCR) measurements were used to verify equilibration of mitochondria to the steady state prior to the start of the different assay protocols. In the following analyses, only the second basal measurement was set as basal OCR. With the Agilent Seahorse technology dissolved oxygen and free protons are measured in a transient microchamber every few seconds over a period of 3 min (6 min after ADP injection), then mean oxygen consumption rate (OCR) is calculated for each measurement and depicted as a single data point. All OCR measurements were performed in technical triplicates for *n* = 6 per group and normalized by equal protein amount of mitochondrial fraction per well and additionally the use of the mitochondrial content marker citrate synthase (CS) activity [33].

For OCR measurement analyses Wave 2.6.0 (Agilent Technologies, Santa Clara, CA, USA) was used. Calculation of RCR required addition of an arbitrary factor to all readings of state 3 and state 4o, to raise negative state 4o values of individual samples to a positive numeric range, so that they could be used for the calculation. The degree of thermodynamic coupling (q) and the optimal thermodynamic efficiencies (*ƞ-opt*) were calculated from state 4o and state 3 values as described elsewhere [34].

Activity of citrate synthase and cytochrome c oxidase was measured with the Citrate Synthase Assay Kit (Sigma-Aldrich/MERCK, Germany) and the Cytochrome c Oxidase Assay Kit (Sigma-Aldrich/MERCK, Germany) according to the manufacturer's protocols, using 10 µg or 60 µg of the 10,000×*g* mitochondrial fraction from five single animals in duplicates, respectively. Membrane integrity was determined from cytochrome c oxidase activity data from six single animals. The enzyme activity of methyltransferases (MTases) and sirtuins (SIRTs; histone deacetylases class III; (SIRT1, SIRT2, SIRT3, SIRT4, SIRT5, SIRT6, SIRT7)) were measured using the MTase Glo Methyltransferase Assay (Promega, Germany) and the SIRT Glo Assay System (Promega, Germany) from 100 ng and 1 µg cell lysates in duplicates from six single animals (600 xg fraction), respectively, according to the manufacturer's protocol. Malondialdehyde (MDA) as a marker for lipid peroxidation was fluorometrically measured from 30 mg of the 600×*g* fraction in duplicates from five single animals using OxiSelect™ TBARS Assay Kit (MDA Quantification) (Cell Biolabs, Inc., USA) according to manufacturer’s protocol.

### Mitochondrial copy number

Genomic DNA was extracted from snap-frozen muscle biopsies using standard technologies (Qiagen, Hilden, Germany). The relative amount of mitochondrial DNA (mtDNA) was determined with gene-specific primers and fluorescent-labeled probes for mitochondrial gene Nd1 and nuclear gene Lpl in triplicates from five animals per group as described [35] by qPCR (Thermo Fisher Scientific, Darmstadt, Germany)

### Proteome analysis

The 10,000×*g* fractions of enriched mitochondria were lysed in loading buffer and subjected to 10% SDS polyacrylamide gel electrophoresis. Coomassie blue-stained protein bands were excised and digested in the gel. The protein-containing gel slices were washed and the proteins were reduced, followed by alkylation. Digestion was performed, and then the eluted peptides were lyophilized. The peptides were reconstituted for mass spectrometry analysis in 1% trifluoroacetic acid (v/v), including index retention time (iRT) peptides (Biognosys, Schlieren, Switzerland). Analyses of the peptides were then performed by LC–MS/MS in a label-free proteome analysis approach with an Ultimate 3000 separation liquid chromatography system combined with an EASY-spray ion source and Orbitrap Fusion Lumos Tribrid mass spectrometer (Thermofisher Scientific, Germany). The peptides were trapped on an Acclaim PepMap C18-LC-column (ID: 75 μm, 2 cm length; Thermofisher Scientific) and separated via EASY-Spray C18 column (ES802; ID: 75 μm, 25 cm length; Thermofisher Scientific). Each LC–MS run lasted 150 min, and MS data were acquired with both data-dependent (DDA) and data-independent (DIA, 34 windows) MS/MS scan approaches. The DDA runs were analyzed using Proteome Discoverer 2.5 software (Thermofisher Scientific) and Sequest HT search (trypsin digestion, max. 2 miscleavages, 5–144 peptide length, max. 10 peptides per spectrum, carbamidomethylation as static and N-terminal acetylation/methionine oxidation as dynamic modifications) against the SwissProt FASTA database (Mus musculus (TaxID = 10,090, version 2021–07)). The percolator node-based peptide-spectrum match (PSM) analysis was restricted to q-values with 0.01 (strict) and 0.05 (relaxed) false discovery rate (FDR). The proteins were filtered using parsimony principle set to 0.01/0.05 (strict/relaxed) FDRs. For quantification of proteins prior to interpretation, the DIA runs virtually collecting all fragmented peptides over the retention time, were analyzed via SpectronautTM Pulsar 15 software (Biognosys, Switzerland) set to standard parameter settings and using a self-performed spectral library based on DDA runs, as described [36]. DIA files were processed using Spectronaut with default settings, (PTM localization activated, PEP cutoff set to 0.75, data filtering set to Q-value, and Normalization Strategy set to Local Normalization). A modified Fisher’s exact test (subtracting one entry of hits) was used for functional enrichment analyses to determine if the enrichment in proteomics observed in one pathway is more than a random chance compared to the mouse background. Z-score significance was assessed for estimation plot analysis using the over and under-representation of proteins in each condition with respect to the overall experimental mean were used. For mitochondrial proteome, five animals per group were analyzed, and the mass spectrometry data are available at the ProteomeXchange Consortium via the PRIDE partner repository with the dataset identifiers PXD035798.

### Transcriptome analysis

Total RNA was extracted with standard procedures from snap-frozen muscle biopsies (Qiagen, Hilden, Germany). Genome-wide expression analysis with *n* = 5 samples per condition was performed using Mouse Gene (MTA) arrays (Thermo Fisher Scientific, Darmstadt, Germany) as described starting from 150 ng total RNA [37]. Datasets are available as super series under https://www.ncbi.nlm.nih.gov/geo/query/acc.cgi?acc = GSE210510 or transcriptome dataset accession number GSE210365. Gene expression data were further analyzed with Transcriptome Analysis ConsoleTM v.4.01 (Thermo Fisher, Darmstadt, Germany), and knowledge-based transcriptome analyses were performed using IPA® (QIAGEN Inc., www.qiagenbioinformatics.com/ products/ingenuity-pathway-analysis) [38], with 1.2-fold differences, p-value < 0.05).

### Methylome analysis

For methylation analysis, genomic DNA was extracted from 20 mg of snap-frozen *M. gastrocnemius* tissue (*n* = 5 animals per condition) using the DNeasy® Blood & Tissue Kit (Qiagen, Hilden, Germany). 500 ng of DNA were shared by ultrasound to an average length of 200 nt (Bioruptor® Pico; Diagenode SA, Seraing, Belgium). DNA was then processed in two steps and subsequently analyzed by Next-Generation Sequencing. First, sample complexity was reduced by enrichment of DNA fragments with methylated cytosines using the EpiXplore™ Methylated DNA Enrichment Kit (Takara Bio Europe, Saint-Germain-en-Lage, France). Next, to facilitate the direct identification of methylated Cs, enriched methylated DNA fragments were subjected to TET-mediated enzymatic conversion of unmethylated cytosines and subsequent library preparation using NEBNext® Enzymatic Methyl-seq Kit (NewEngland BioLabs® Frankfurt, Germany) according to manufacturer's instructions. Libraries were paired-end sequenced on the NextSeq550™ instrument with automated FastQ raw data analyses (Illumina, San Diego, USA). Data are available as superseries or methylome data at NCBI GEO https://www.ncbi.nlm.nih.gov/geo/ dataset accession GSE210509).

For methylome analyses, raw Illumina paired-end reads were converted into FastQ format by executing bcl2fastq2 Conversion Software version 2.20 (Illumina, San Diego, USA). FastQ files of methylation analyses were analyzed using the Illumina Dragen® (Dynamic Read Analysis for Genomics) Bio-IT platform v 3.9.5 (Edico Genome, Illumina San Diego) on the Illumina cloud providing predesigned analysis pipelines. Runs were prepared and quality checked using Fast Q toolkit pipeline (v1.0.0.) with a minimum length of 32 bp adapter trimming using the TRUESEQ HT/LT adapters sequence (Illumina San Diego, USA). Settings were: trimming strength: 4 and a quality score < 10 to trim bases at 5’ and 3’ sites before mapping. Paired read FastQ files were then mapped using a Hash table generated by DRAGEN Reference builder (v.3.10.4) based on the mouse genome (GRCm38.p6.genome.fa, including methylation information). Mapping was performed using the DRAGEN Methylation pipeline (version v3.9.5) based on [39] for read conversion (C-to-T and G-to-A), deduplication, sorting, and alignment of bisulfite converted reference genome, genome-wide methyl calling and the calculation of alignment and methylation metrics. The outputs of sequence alignment/map files were further analyzed using the R package MethylKit [40] (BaseSpaceLabs, Illumina San Diego, USA). CpG sites with a minimum read quality of at least 20 and a minimum read coverage of at least 5, as well as a maximum coverage of 99.9%, were considered for differential methylation analysis with > 2% difference in conditions. In the single-base CpG resolution analysis, methylation calls were optimized by combining methylated sites from both the forward and reverse reads. Settings were as: 5 × CpG Coverage, 2% methylation difference, and q value of 0.05).

### Statistics

For non-OMICS analyses, significant differences between means for variables displaying normal distribution were determined using unpaired two-tailed Student t tests for two variables or one-way ANOVA with Tukey test for more than two groups. A Shapiro–Wilk analysis was used to confirm that the sample were normally distributed. Similarly, variables that did not follow a normal distribution were analyzed with Mann–Whitney test, when appropriate. P values are corrected for multiple testing using the Benjamini–Hochberg (FDR) method**.** Data are presented as the mean ± 95% confidence interval (CI) unless otherwise stated. All calculations and graphs were made using GraphPad Prism software version 9.4.0 (GraphPad Software Inc., La Jolla, USA).

## Results

### Metabolic characteristics after Cvs

12-week-old male C57BL/6 mice were subjected to our 15-day stress protocol, whereas control mice were kept untreated. The comparison of body weight before and after the stress intervention showed a significant decrease of up to 2 g in the stressed animals, whereas the control animals showed an increase of 4 g in body weight (Fig. 1A). Quantification by NMR revealed a significant change in body composition. In detail, in this short phase stress decreased not only the fat mass up to 10% (approx. 0.4 g) but also the lean mass up to 7.5% (approx. 2 g) (Fig. 1A). In contrast, the control animals showed a significant increase in fat mass of up to 10% (approx. 0.3 g) (Fig. 1A) and lean mass of 7.5% (approx. 2 g) (Fig. 1A).

The weight change of the animals during the Cvs protocol was not due to food intake. During the stress intervention, the Cvs animals had a 20% higher average energy intake per gram of body weight compared to the control animals (Fig. 1B). Consistent with this, body weight changes per convertible energy (mg/kJ) were significantly different and showed a decrease in the Cvs group compared to controls (Fig. 1B).

### Indirect calorimetry in Cvs mice

**Fig. 2:**
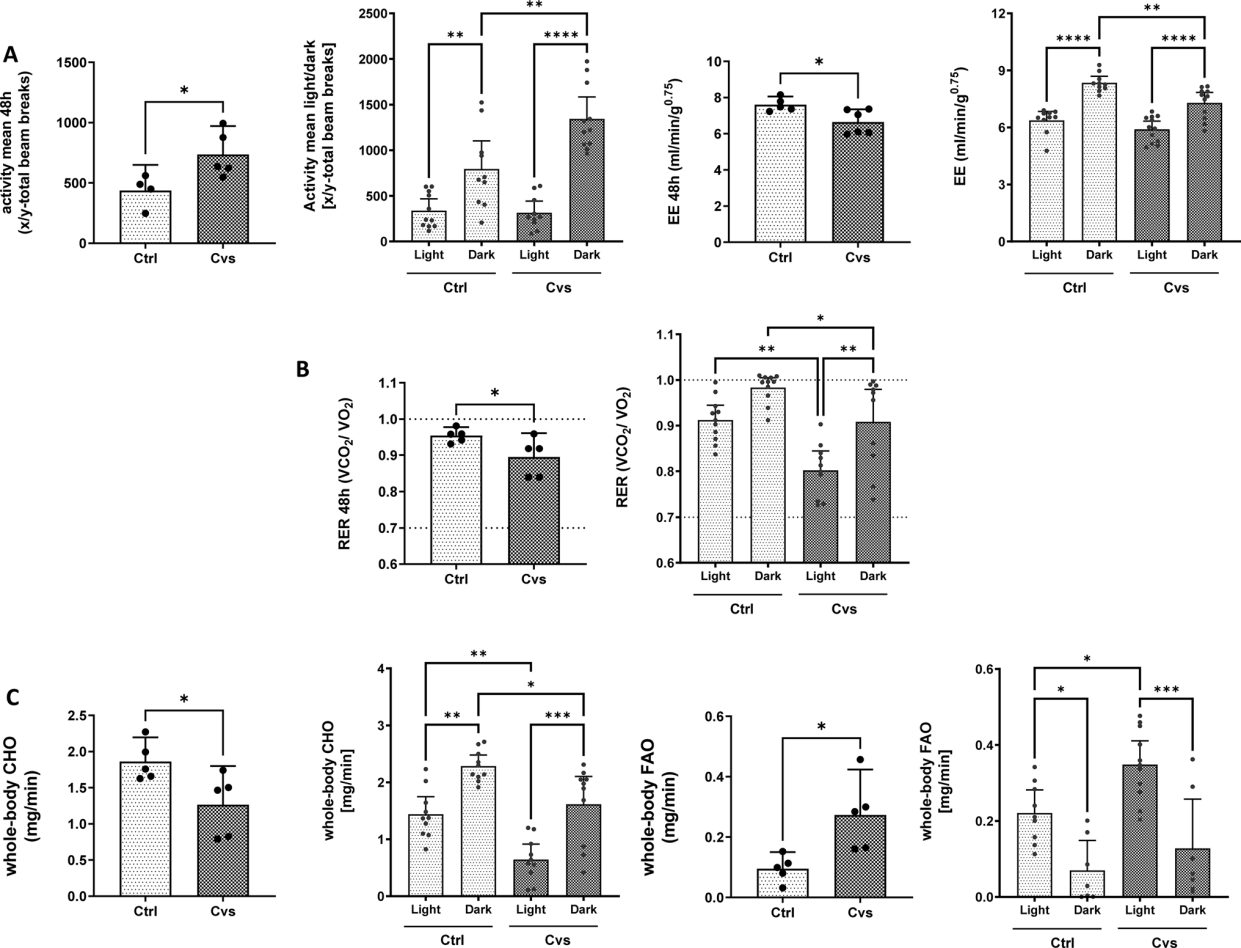
Effect of 15 days of Cvs intervention on physical activity, energy expenditure (EE), and substrate utilization. **A** Detailed differences in activity and energy expenditure (EE) of Cvs mice in comparison to the Ctrl group during light and dark phases, **B** Respiratory exchange ratio (RER). The dotted lines mark the thresholds for preferential whole-body carbohydrate oxidation (CHO) (VCO_2_/VO_2_ = 1.0) or whole-body fatty acid oxidation (FAO) (VCO_2_/VO_2_ = 0.7) substrate utilization, (**C**) CHO and FAO. The pairwise bar graphs represent the mean ± 95% CI over 48 h measurement, dots indicate single animals (*n* = 6/group). Graphs separated for light and dark phases depict each light or dark phase measurement for the respective animal (*n* = 6/group, with two measurements per phase). Statistics: two-tailed paired *t* test or one-way ANOVA with Tukey test for multiple comparisons, *p < 0.05, **p < 0.01, ***p < 0.001, ****p < 0.0001, *Ctrl* C57BL/6, *Cvs* C57BL/6 after stress intervention, *CHO* carbohydrate oxidation, *FAO* fatty acid oxidation

To investigate the differences observed, the metabolic activity was measured by indirect calorimetry immediately after Cvs. Data were analyzed for light and dark phases to distinguish metabolic phenomena, depending on low (light) and high (dark) activity levels. Compared with control mice, the stressed mice showed an increase in total physical activity over the observation period, which is especially due to increased dark phase activity (light: Cvs = 389; Ctrl = 288; dark: Cvs = 1245; Ctrl = 794) (Fig. 2A). Total energy expenditure (EE) during the entire observation period was significantly lower after Cvs compared to control animals (Cvs = 6.3 ml/min/g*^0.75^; Ctrl = 7.2 ml/min/g*^0.75^) (Fig. 2A). Consistent with higher dark phase physical activity the circadian EE indicated in all animals that the EE rates of the dark were significantly higher (7.9–8.3 ml/min/g*^0.75^) than light period (5.9–6.3 ml/min/g*^0.75^). The overall decrease in EE in stressed mice was solely due to the reduced dark EE (Fig. 2A).

Over the observation period, respiratory exchange ratio (RER) indicated a shift in whole body substrate utilization from preferential carbohydrate utilization towards fat oxidation in Cvs mice (Ctrl = 0.95; Cvs = 0.89). In stressed animals, the RER was significantly decreased compared to control mice in both, the light and dark phases, (Fig. 2B), with the greatest decrease in the light phase. Detailed analyses showed that carbohydrate oxidation (CHO) was reduced after Cvs (Fig. 2C). In both groups, CHO was mainly driven in the dark phase (Ctrl: 2.2 mg/min; Cvs: 1.4 mg/min) in contrast to the light phase (Ctrl: 1.4 mg/min; Cvs: 0.6 mg/min) and the Cvs group exhibited a significantly decreased circadian CHO compared to controls (Fig. 2C). In contrast, the average fat oxidation (FAO) increased after Cvs. In both groups, FAO was higher in the light phase than in the dark phase. The observed difference comes from a significantly increased FAO during light phase in Cvs mice (Fig. 2C). So, the Cvs group showed an overall decrease in whole-body substrate utilization, except for resting FAO during light phase.

### Mitochondrial composition assessment

**Fig. 3:**
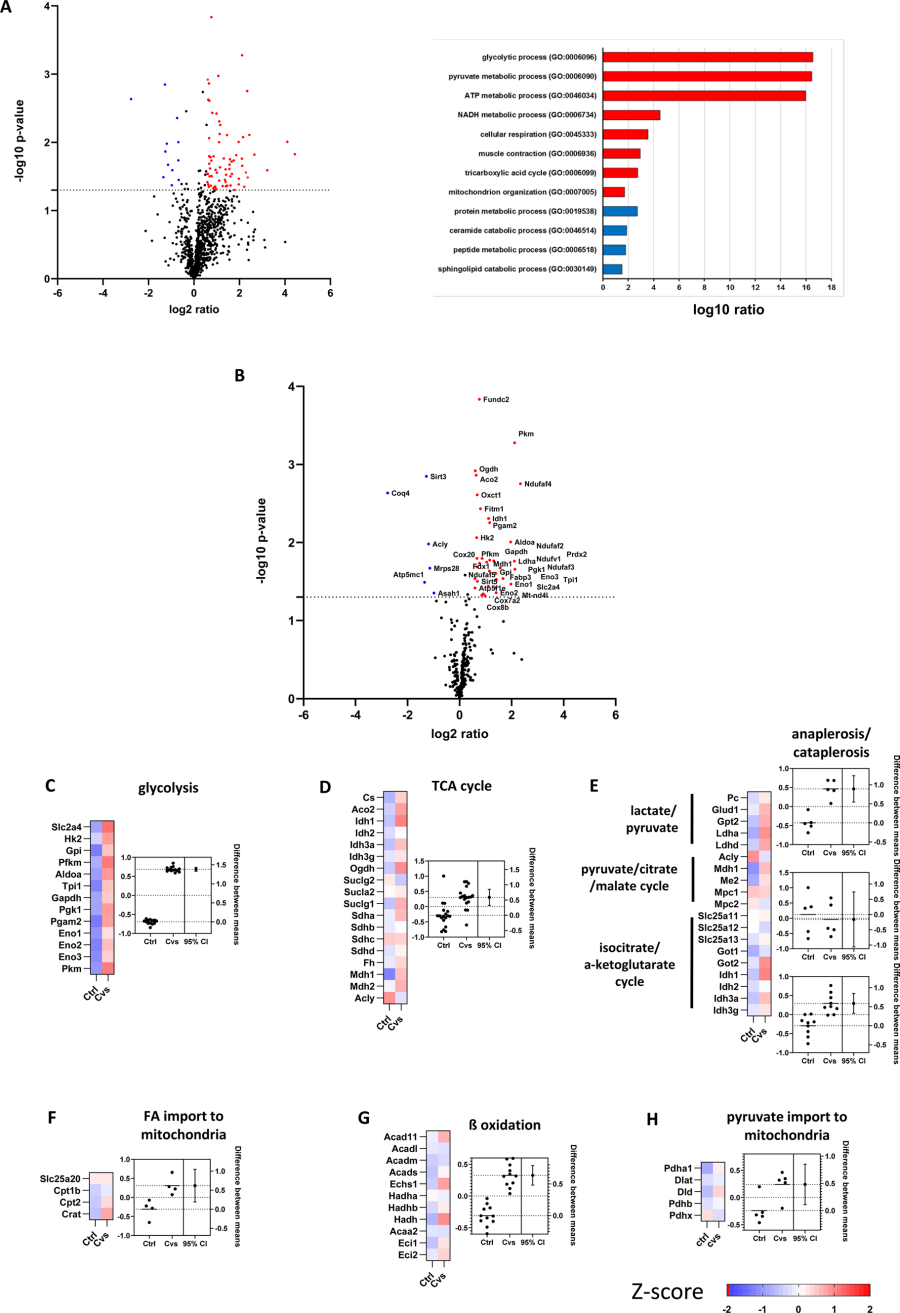
Cvs intervention interferes with metabolic pathway component abundance in enriched muscle mitochondria. **A** Volcano plot analyses and Gene Ontology (GO) classification of proteome analyses. Volcano plot analysis reveals the significant 73 up-regulated (red) and 13 down-regulated (blue) proteins from label-free proteomic analysis comparing Ctrl and Cvs group (*n* = 5 / group). X-axis shows log2-ratio and Y-axis shows − log10 *p*-value with the dotted line representing the threshold value of statistical significance (student's *t*-test (*p* < 0.05); > 1.5-fold regulation). Significantly enriched pathways (FDR < 0.1) are shown for the GO ontology for biological processes. Functional enrichment was determined with a modified Fisher’s exact test with Benjamini–Hochberg (FDR) for multiple testing correction. **B** Volcano plot analyses of ETC, mitochondrial and associated pathways derived from proteome analyses. Volcano plot analysis shows the significant 34 up-regulated (red) and 6 down-regulated (blue) proteins corresponding to the analyzed metabolic pathways and ETC from label-free proteomic analysis when comparing the Ctrl and Cvs groups (*n* = 5 / group). The significant proteins are shown here with their respective names. X-axis shows log2-ratio and Y-axis shows -log10 p-value with the dotted line representing the threshold value of statistical significance (student's *t*-test (*p* < 0.05); > 1.5-fold regulation). **C–H** Z-score plots show the over and under-represented proteins of the indicated pathways. Red identifies upregulation, blue identifies downregulation, and white indicates no change of protein abundance to the mean of each condition with respect to the overall experimental mean. The corresponding estimation plots show on the left axis scatter dot plots with mean z-scores, while dots represent each pathway protein as mean of *n* = 5/ group. On the right axis the mean ± 95% CI alteration in pathway protein abundance of the Cvs and Ctrl comparison is shown. Dotted lines represent the mean of each group centered on 0. **C** Glycolysis, **D** TCA cycle, **E** ana / cataplerosis **F** fatty acid import into mitochondria, **G** ß-oxidation (mitochondria), **H** pyruvate import into mitochondria. *ETC* electron transport chain, *TCA* tricarboxylic acid cycle, *Ctrl* C57BL/6, *Cvs* C57BL/6 after stress intervention

To follow this, we analyzed how stress interferes with the *status quo* of cellular energy metabolism in a label-free proteomic analysis of 10,000×*g* fractions. Following stress, mitochondrial DNA copy number determined by mtDNA/nDNA ratio showed a small but significant increase in the muscles of the stressed mice (Supplement Fig. 2A). In the enriched mitochondrial fractions, mitochondrial content, as determined by the commonly used valid biomarker citrate synthase activity [41], was not significantly altered within the groups (Supplement Fig. 2B). Mitochondrial membrane integrity analysis, based on cytochrome-c-oxidase activity, showed an average integrity of above 90% with no differences between both groups (Supplement Fig. 2C). In the label-free proteomic analysis, 1090 proteins were identified. Of these, 86 proteins were significantly altered in abundance in Cvs mice (Up-regulated: 73/ Down-regulated: 13; ratio: > 1.5; *p*-value: < 0.05) (Fig. 3A) (Supplement table 1). Gene ontology analysis of biological processes indicated changes in catabolic processes. In the more detailed analysis of the proteome, the metabolic pathways and the ETC were examined separately and revealed 34 up-regulated and 6 down-regulated proteins followed Cvs (Fig. 3B). The most prominent change with significantly increased protein abundance was the glycolysis pathway (Fig. 3C). In contrast, key catabolic pathways, like ketolysis, glutaminolysis, and branched-chain amino acids metabolism were unaltered by Cvs (Supplement Fig. 3). The proteome data pointed to increased mitochondrial activity, as tricarboxylic acid (TCA) cycle and the ana/cataplerosis pathway proteins showed higher abundances after Cvs (Fig. 3C, D). In addition, fatty acid import into mitochondria, ß-oxidation, and pyruvate import into mitochondria was elevated after stress intervention (Fig. 3E–H).

**Fig. 4:**
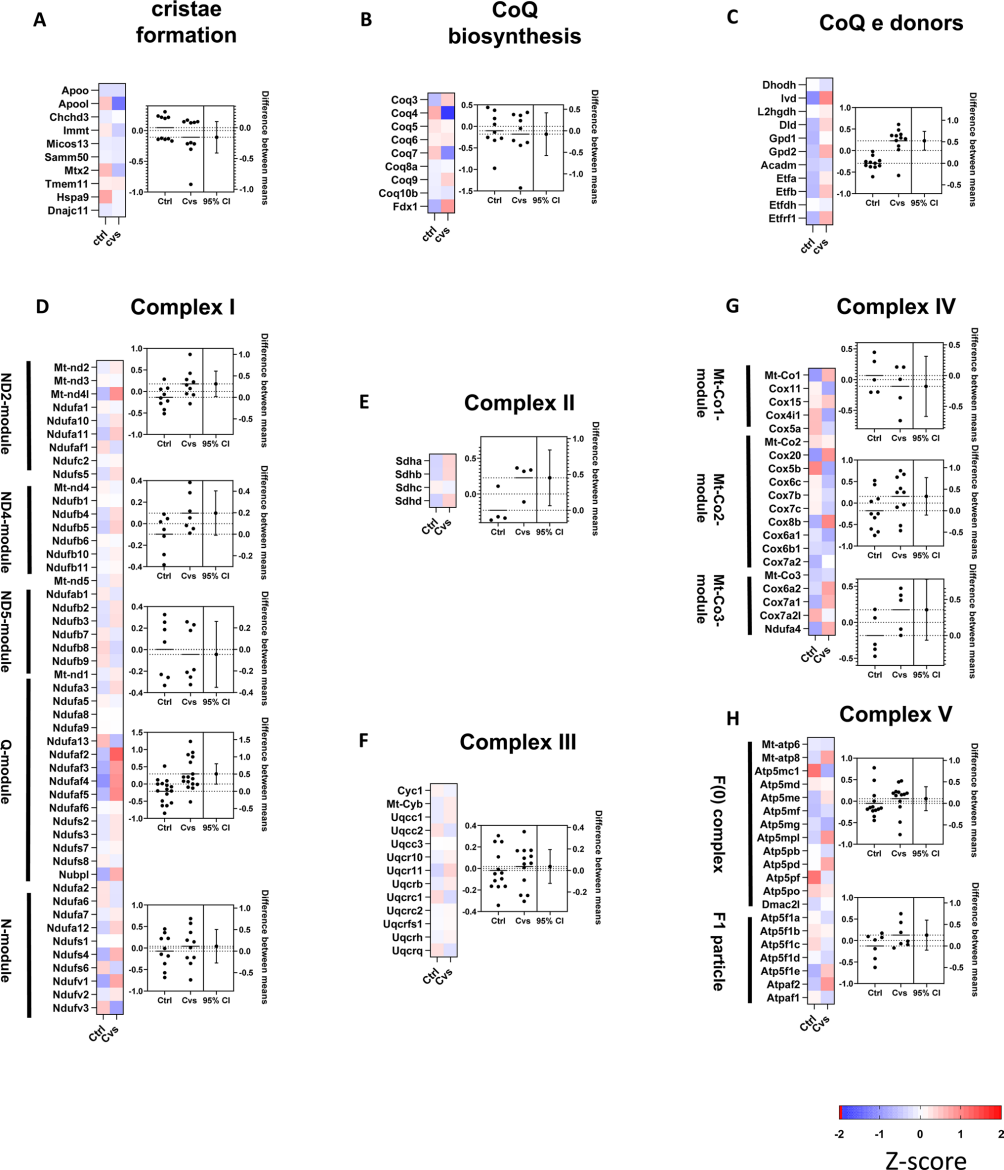
Mitochondrial proteome. **A–C** Z-score plots show the over and under-represented proteins of the indicated pathways. Red identifies upregulation, blue identifies downregulation, and white indicates no change of protein abundance of the mean of each condition with respect to the overall experimental mean. The corresponding estimation plots show on the left axis scatter dot plots with mean z-scores, while dots represent each pathway protein as mean of *n* = 5/ group. On the right axis the mean ± 95% CI alteration in pathway protein abundance of the Cvs and Ctrl comparison is shown. Dotted lines represent the mean of each group centered on 0. Plots resulting from z-score analyses and estimation plots: **A** cristae formation, **B** CoenzymeQ (CoQ) biosynthesis, and (**C**) CoQ e donors, **D–H** Z-score analyses of protein abundance for individual complex I, II, III, IV, and V subunits of electron transport chain. *Ctrl* C57BL/6, *Cvs* C57BL/6 after stress intervention

The proteome data after the Cvs period pointed towards an increase in mitochondrial key metabolic protein components to maintain energy supply. Next, we assessed mitochondrial component compositions about protein abundance differences in sum indicated by estimation plots. There were no significant changes in proteins involved in cristae formation following Cvs (Fig. 4A), and the Coenzyme Q (CoQ) biosynthesis modules (Fig. 4B). The CoQ e donors, i.e. the additional respiratory membrane-bound complexes, showed increased abundances after Cvs (Fig. 4C). A total of 109 of identified proteins could be assigned to the electron transport chain (ETC) (Fig. 4D–H). Complex I and III were unaltered (Fig. 4D, F) in muscle mitochondria derived from Cvs animals. Complex II showed increased abundance between the Cvs and Ctrl groups (Fig. 4E). Complex IV showed differences in subunit abundance of the Mt-Co2 and the Mt-Co3-modules, while Mt-Co1 remained unchanged after stress intervention (Fig. 4G). Although single proteins of complex V F(0) complex and F1 particle were altered, there was no significant overall change (Fig. 4H). In total, the analysis of protein composition of the ETC complexes I to V showed only few changes in single subunit abundances, suggesting that Cvs intervention did not induce substantial changes in ETC composition.

### Cvs interferes with mitochondrial function and EE in muscle

Alterations in fuel preferences and the composition of major mitochondrial metabolic pathways, without evidence of modulation of ETC complex proteins or changes of mitochondrial structural components for cristae formation, may indicate altered mitochondrial activity after Cvs. In addition, the abundance of UCP3 at the transcriptional and protein levels was not altered between the two groups (Supplement Fig. 4 A, B). The status of oxidative stress was assessed by proteome analyses of redox-regulating proteins and by measuring the reaction of malondialdehyde with thiobarbituric acid. Here, the proteome and enzyme analysis indicated no changes in redox balance between Cvs and Ctrl groups (Supplement Fig. 4C).

**Fig. 5:**
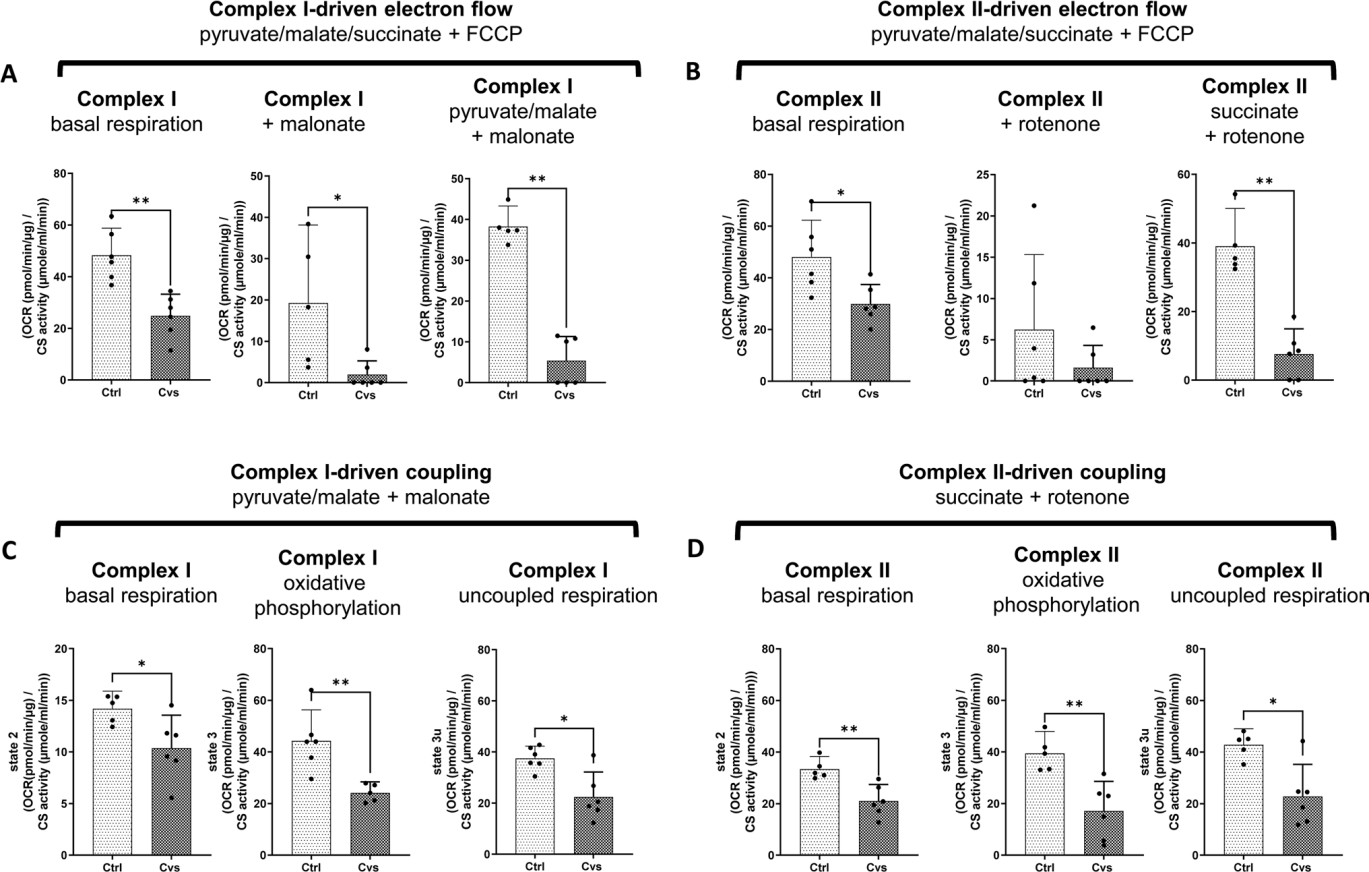
Electron flow and coupling experiments of mitochondrial ETC after Cvs. Respiratory capacity was measured in the enriched mitochondrial fractions of isolated muscle mitochondria from Cvs compared with Ctrl muscles in response to ETC manipulation. **A**, **B** Results from electron transport measurements in uncoupled mitochondria (induced by FCCP) specific for complex-I (**A**) and complex II (**B**) –driven electron transport. Oxygen consumption rate (OCR) at basal level was measured in the presence of unlimited substrate condition (pyruvate/malate/succinate), complex-specific OCR was measured after inhibition of the respective other using malonate or rotenone and stimulated complex-specific OCR was measured after addition of fresh complex specific substrate, namely pyruvate/ malate or succinate (**C**) Complex I-specific coupling experiment. OCR was measured using complex I-specific substrate pyruvate and malate, with inhibition of complex II activity by malonate. **D** Complex II-specific coupling experiment. OCR was measured using complex II-specific substrate succinate, with inhibition of complex I activity by rotenone. (**C/D**) Basal respiration (state 2), oxidative phosphorylation (state 3), and maximal uncoupled respiration (state 3u) measures are shown for the complex I- and complex II-specific assays. The OCR values for all experiments were normalized by citrate synthase (CS) activity to adjust for changes in mitochondrial content. Bar graphs represent the mean of *n *= 6 animals/ group ± 95% CI. Individual measures for each animal are given as dots. Statistics: Mann–Whitney test, *p < 0.05, **p < 0.01, *Ctrl* C57BL/6, *Cvs* C57BL/6 after stress intervention. Electron flow and coupling experiment OCR time courses are given in Supplement Fig. 5A, B

First, to determine potential differences in substrate entry to the ETC, and eventual differences in the capacity of such entry on electron transport, basal oxygen consumption rate (OCR) was measured without substrate limitation in uncoupled mitochondria. This was followed by subsequent inhibition of complex I or II using malonate or rotenone in two different experimental setups, respectively, with subsequent complex-specific stimulation of the respective other either by pyruvate/ malate or succinate (Supplement Fig. 5). In both settings, the Cvs group showed significantly decreased OCR values over the time course of the assay (Supplement Fig. 5). Already the basal level in the presence of all substrates to drive complex I as well as complex II in the uncoupled condition, showed lower OCR values (46% for complex I; 38% for complex II-driven respiration) after Cvs compared with the control group (Fig. 5A/B). Inhibition of complex II under these conditions decreased respiration rate by half in both groups and the complex I-specific electron transport showed a fourfold decrease in OCR in mitochondria following Cvs compared with control animals (Fig. 5A). Inhibition of complex I by rotenone showed a strong reduction in OCR in both groups (Fig. 5B). Forced electron flow either through complex I or II by additional pyruvate/malate or succinate addition in uncoupled Cvs mitochondria resulted in significant fourfold reduction of OCR compared to controls (Fig. 5A/B).

To follow the complex I and complex II-independent reduction in electron flows after Cvs, the coupling efficiency of ETC complexes I to IV to ATP synthase (complex V) were measured. Complex I-driven respiration was forced with the specific complex I substrates pyruvate/malate with simultaneous inhibition of complex II by malonate and was significantly lower after Cvs (Fig. 5C; Supplement Fig. 5B). Detailed examination of complex I-driven mitochondrial function revealed a 40% lower OCR in state 2 in Cvs animals compared to controls (Fig. 5C). The initiation of oxidative phosphorylation (state 3) with ADP showed a significant 55% decrease in OCR in the Cvs animals (Fig. 5C). Here, state 3 via complex I, significantly correlated with interindividual light phase EE rates (*R*^2^ = 0.405; *p*-value = 0.0261), but not with the dark phase EE. Maximal uncoupled respiration (state 3u) after FCCP injection was significantly decreased by 42% in mitochondria from Cvs mice (Fig. 5C) but showed no correlations to circadian EE rates.

Complex II-specific coupling forced by the specific substrate succinate and simultaneous inhibition of complex I by rotenone revealed a consistently lower OCR over time in Cvs mitochondria compared to control (Fig. 5D). Complex II-driven function showed a 36% lower respiration in state 2 and 56% lower respiration in state 3 in the Cvs group (Fig. 5D), both without correlations to circadian EE rates. The state3u via complex II (state 3u) showed a 47% reduction following Cvs, and there was a significant intraindividual correlation to both, light and dark EE (light: *R*^2^ = 0.549; *p*-value = 0.0091/ dark: *R*^2^ = 0.432; *p*-value = 0.0278). Overall, states 3 and 3u were reduced for both complex I- and complex II-driven coupling after chronic stress.

### Bioenergetics assessment of mitochondria by respiratory control ratio and calculation of thermodynamic coupling and efficiency

**Fig. 6:**
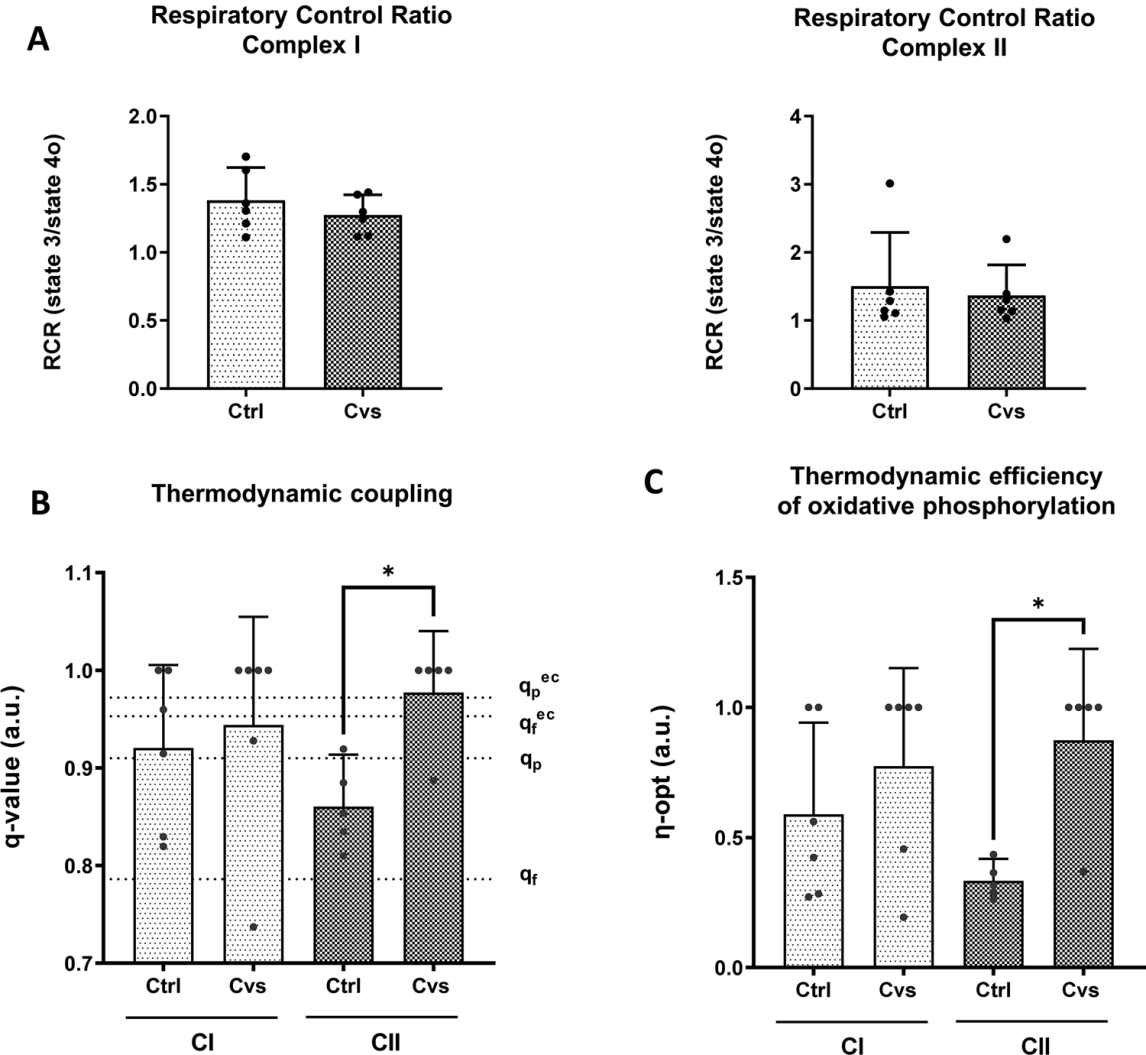
Respiratory control ratio, thermodynamic q value and calculated optimal thermodynamic efficiencies of mitochondrial complex I and II after Cvs. Respiratory control ratio (RCR) was calculated as state 3/state 4o from complex I- and complex II-specific respiration (*n* = 6 animals/group). **B** Calculated thermodynamic coupling q values are shown for complex I- and complex II-dependent respiration. Dotted lines indicate the maximal coupling values of the thermodynamic set points corresponding to maximal net output flow (ATP) at optimal efficiency (*q*_f_ = 0.786), maximal net output power (*q*_ϱ_= 0.910), economic net output flow (*q*_f_^ec^ = 0.953), and economic net output power at optimal efficiency (*q*_ϱ_^ec^ = 0.972). (*n* = 6 animals/ group). **C** Calculated optimal thermodynamic efficiencies (*ƞ-opt*) of oxidative phosphorylation are shown for complex I- and complex II-dependent respiration. Data are expressed as means ± 95% CI, the dots represent single values for each animal. Statistics: Mann–Whitney test and one-way ANOVA with Tukey test for multiple comparisons, *p < 0.05, *Ctrl* C57BL/6, *Cvs* C57BL/6 after stress intervention

For the assessment of the quality of mitochondrial activity, the respiratory control ratio (RCR), a reference value for proton leak was calculated (state 3/state 4o). The Cvs RCRs for neither complex I- nor complex II-driven respiration differ from the controls (Fig. 6A). To determine the thermodynamic coupling of oxidative phosphorylation capacity the *q-value* was calculated (Fig. 6B). This approached the thermodynamic set point of economic net output power at optimal efficiency. These analyses define the maximal net output flow (ATP) at optimal efficiency (*q*_f_ = 0.786), maximal net output power (*q*_ϱ_ = 0.910), economic net output flow (*q*_f_^ec^ = 0.953), and economic net output power at optimal efficiency (*q*_ϱ_^ec^ = 0.972) [34]. The oxidative phosphorylation capacity of complex I was unaltered by Cvs. For control animals, the coupling of complex I (> stage *q*_p_ (= 0.910)) is greater than that for complex II-driven respiration (< stage *q*_p_ (= 0.910)). The complex II oxidative phosphorylation capacity was significantly enhanced after Cvs compared to controls (Fig. 6B). Moreover, for stressed animals, the coupling of complex II was greater (> stage *q*_p_^ec^ (= 0.972)) than that for complex I-driven respiration (< stage *q*_f_^ec^ (= 0.953)). In accordance with that, the efficiency of substrate to energy conversion (*ƞ-opt*) related to the thermodynamic coupling shows significantly higher levels in Cvs mitochondria with an increase of up to 38.6% compared to the Ctrl group (Fig. 6C).

### Cvs effects on muscle transcriptome and methylome

According to our hypothesis, Cvs interferes directly with metabolism but also initiates a memory effect for metabolic adaptation. The genome-wide DNA methylation patterns in *M. gastrocnemius* showed a tight correlation between the conditions and thereof no gross alteration directly after chronic stress in muscle (Supplementary Table 2; Supplementary Fig. 6). The only significant variations found on methylation level were the hypomethylation of three intergenic areas including some coding genes like neuroactive Gphn, DDscaml, cell cycle active Sdk1, Mid1, and the nuclear-encoded mitochondrial leucyl-tRNA synthetase Lars2, but methylation differences were low (Supplementary Table 2). Consistently, there were no changes in Sirt and MTase activity (Supplementary Fig. 7). In concordance, there was no gross significant alteration in transcriptome level maintained after the stress phase within Cvs and Ctrl mice even for 1.2-fold expression differences (*p*-value < 0.05) (Supplementary Table 3; Supplementary Fig. 8). Nevertheless, ND1R1 and 6 mitochondrial tRNAs (mt-Tc, mt-Tt, mt-Tf, mt-Tl2 and the mitochondria-specific iso-acceptors mt-Ts1, mt-Ts2) were in the 10 most suppressed transcripts after Cvs. Mitochondrial tRNAs differ from nuclear tRNAs in sequence, stability, and folding, and are essential for mitochondrial protein synthesis. This may indicate alterations in the translation of mitochondrial-coded transcripts, and may additionally point to the central role of mitochondrial function after Cvs also in the long-term alterations.

## Discussion

In the present study, we investigated the acute and adaptive effects of chronic variable stress (Cvs) on energy metabolism in skeletal muscle as the main determinant of whole-body energy expenditure. Analyses of the transcriptome, methylome, and proteome, as well as mitochondrial function in Cvs mice, were compared to controls. We show, that immediately following Cvs (i) body weight, fat- and lean mass decreased despite higher food intake, (ii) nocturnal energy expenditure (EE) is lower despite higher activity, (iii) respiratory exchange ratio (RER) shifted from carbohydrate (CHO) to fatty acid oxidation (FAO), (iv) mitochondrial metabolic capacity was altered with changes in the mitochondrial proteome, and (v) mitochondrial coupling capacity was reduced, but the thermodynamic efficiency increased.

The advantage of preclinical animal models over human studies is the tight control of the environment by a standardized laboratory and the uniform genetic background of used mice. Thus, the Cvs effect can be isolated from interaction with exogenous confounding factors. With this, analyses of Cvs in a standardized preclinical model may help to unravel even metabolic fine-tuning effects of cellular adaptation to maintain energy balance, which may be superimposed by confounding effects in more complex and externally influenced systems as clinical studies. Consistent with previous studies, Cvs exposure resulted in immediate activation of the hypothalamic–pituitary–adrenal (HPA) axis [4], with high corticosterone levels, and increased metabolic risk factors such as higher fasting blood glucose, triglycerides, and NEFA levels in plasma. These complex stress-induced alterations are consistent with the well-known pleiotropic effects of corticosterone on glucoregulatory insulin-responsive tissues [42]. So, the system used here mirrors human stress and chronic glucocorticoid (GC) exposure as risk of metabolic syndrome [1–3, 5, 26, 27].

Stress can interfere with food-seeking behavior, including high fat and sugar intake inducing obesity or alcohol-intake interfering with neuropsychiatric function and regulation of satiety hormone action as a futile cycle that may result in a dysfunctional HPA axis [43, 44]. As a result of these complex and integrative processes, GC excess, either endogenous (e.g. Cushing’s syndrome) or exogenous, remodels body composition with central obesity and muscle atrophy [45]. In a mouse model of Cushing syndrome insulin sensitivity and metabolic parameters negatively correlated to the loss of *M. gastrocnemius*, not *M. soleus*, mass [46]. Here, we observed a decreased lean and fat mass with a reduction in circadian EE despite increased food intake.

Furthermore, Cvs shifts energy utilization towards a greater proportion of FAO in whole-body substrate oxidation, despite relatively carb-heavy standard feed. The Cvs effect is mainly based on increased FAO during the light phase, while CHO is reduced during rest phase, as well as CHO and FAO during the active dark phase. The data may suggest that energy is restored after Cvs intervention by whole-body FAO during periods of low activity to compensate for reduced EE during the active phase. Lipid storage in white adipose tissue (WAT), remodeling or browning of WAT, and uncoupled mitochondrial respiration to thermogenesis in brown adipose tissue (BAT) also mediate systemic influence on FA metabolism and EE. Stress can trigger norepinephrine-mediated ß-adrenergic receptor activation in BAT and WAT, promoting cAMP-mediated lipolysis, browning of WAT, and thermogenesis in BAT [47–49]. Thus, increased lipolysis can provide the FA requirement after Cvs, which is reflected in the decreased fat mass. Also cold exposure is one major driver for thermogenesis in BAT and repetitive periods of even short-term exposure increases total EE coupled to the expression of Pgc1 and Ucp1 in BAT [50]. Even though cold exposure is a component of our Cvs protocol, EE was decrease in our mice after Cvs, so thermogenesis of BAT may not be the primary process, here. In addition, BAT is an endocrine organ and intraorgan crosstalk e.g. with muscle has been shown [51, 52]. Batokines such as FGF21, regulating the expression of thermogenic genes, or IL-6 can be secreted in response to stress [47, 51, 53, 54]. Of note, consistent with observations after acute cold stress [53], previous studies using our stress protocol showed that in the experimental timeframe immediately after Cvs, serum levels of FGF21 and IL-6 were not significantly altered [26, 27].

However, from our previous work, we saw no differences in glucose- and lipid uptake and insulin signaling in muscle tissues in our Cvs model [26]. Although muscle FAO alone may not account for the changes in whole-body EE, there were signs of increased mitochondrial capacity that might affect muscle mitochondrial function. In addition, in the present analyses, proteins of the ana-/ catabolic pathways are increased following stress, which implicates an increased metabolic turnover after Cvs.

The enrichment analysis of the differentially expressed proteins revealed that next to strictly mitochondrial proteins also others including metabolic-relevant proteins were enriched, which is consistent with previous reports [55]. Here, the proteome analyses revealed changes in metabolic key enzymes in muscle tissue after Cvs. The rate-limiting enzymes of the glycolysis pathway hexokinase, phosphofructokinase, and pyruvate kinase significantly increased in abundance after Cvs. Also the abundance of 3-oxoacid CoA-transferase 1 of the ketolysis pathway, isocitrate dehydrogenase of the TCA cycle pathway and the glutamic-oxaloacetic transaminase of the glutaminolysis pathway were significantly increased in Cvs muscle. These changes in protein data suggests upregulation of catabolic processes in the *M. gastrocnemius* after chronic stress intervention.

Mitochondrial dynamics including movement, tethering, fusion, and fission events are important for cell viability, senescence, intracellular signaling, mitochondria health, and bioenergetics function [56]. It still remains open if changed ATP demand or supply due to the balance of environmental influences and mitochondrial capacity are cause or consequence of the dynamic processes. However, Cvs may influence this process. Based on our proteomic data central proteins for fusion (Mfn1, Mfn2, as well as the Opa1 protein [56, 57]) or fission (Fis1, Mff, Drp1) and associated recruiting factors (MiD49 and MiD51 [58, 59]), if present in the dataset were unaltered by Cvs. Depending on the circadian rhythm, Cvs causes a greater fluctuation in substrate utilization shown by decreased EE and CHO to save substrates in the activity phase, and recovery of energy demands in resting times by unaltered EE, accompanied by preferential FAO. In addition, we observed a correlation between energy expenditure and mitochondrial capacity. However, there was no evidence for oxidative stress, as ROS production even tended to be lower after Cvs. Taken together, this suggests an imbalance of biogenesis to degradation, which is attempted to be compensated by the mitochondria as well as fuel utilization.

The gradual decline of mitochondrial functionality from reversible to irreversible physiological defects is a key event in metabolic diseases. This is due to the fact that mitochondria orchestrate energy homeostasis up to all levels of gene regulation. Mitochondria are the first responders to changing conditions and are an important organelle in triggering catabolic processes that affect cellular homeostasis, as seen here after Cvs. The gradual process in mitochondrial adaptation to altered metabolism follows a sequence from functional alteration, functional impairments with compensatory mitochondrial and genomic gene expression, and mitogenesis, up to organelle exhaustion and mitophagy [18, 20, 21].

GC administration can improve skeletal muscle mitochondria respiration even in a mouse model for Duchenne Muscular Dystrophy [60]. Therefore, the differences in EE and RER seen after Cvs were most likely due to mitochondrial function itself. Defects in mitochondrial oxidative phosphorylation and lower mtDNA copy number are defined risks in diabetes type 2 patients [61]. Clinically, it has been shown that primary mitochondrial dysfunction due to mutations in genes encoding mitochondrial proteins can cause defects in energy metabolism and this may account for GC action directly [4, 18, 41, 62]. As mtDNA is not directly correlated to mitochondrial content [41], an increased mtDNA content in Cvs skeletal muscle compared to Ctrl suggests that compensatory processes have already begun at this stage. Our observation is consistent with previously reported stress and corticosteroids regulation of mitochondrial DNA content and gene expression [18, 19]. In enriched mitochondrial fractions with comparable mitochondrial content, the mitochondrial proteome composition of *M. gastrocnemius* shows only a few changes in the electron transfer chain (ETC) components after Cvs. There were no hints to structural alterations, key proteins involved in mitochondrial dynamics fission or fusion in the mitochondrial proteome data, although the system used is able to detect such alterations [63]. However, this interpretation is implicative, as morphological alterations of the mitochondria were not determined in the present study. So, the shift in fuel preferences and altered EE could not be explained by reduced lean mass or changes in relevant mitochondrial structural proteins, which may indicate modifications in mitochondrial activity after Cvs.

The respiratory profile of muscle mitochondria after and the ability of the ETC to transfer electrons were analyzed in isolated mitochondria of muscle tissue after Cvs. Since the assessment of mitochondrial morphology is implicit based on only proteome analyses, respiration data were normalized by defined protein input and by the accepted marker of mitochondrial content citrate synthase activity [41, 64]. Since both normalization strategies have limitations [33, 65], both approaches were used in combination for our purposes to avoid possible normalization-related misinterpretations. So, the presented results show Cvs effect that is independent of potential alterations in total protein content of enriched fractions or mitochondrial quantity based on citrate synthase activity.

The respiratory profile of isolated muscle mitochondria is reduced via both, complex I or II after Cvs. Our results agree with significantly decreased mitochondrial complex I enzyme activity data obtained in patients after corticosterone treatment [66, 67]. Moreover, we found that maximal uncoupled respiration (state 3u) of complex II correlates with whole-body EE Since skeletal muscle is a major contributor to total EE in vivo, this suggests that total EE is dependent on mitochondrial function and coupling. Therefore, it appears that there is a specific mitochondrial impairment involved in GC-induced muscle atrophy [68]. In addition, we observe a reduced electron flow accompanied by significantly decreased mitochondrial coupling independent of NADH (complex I)-linked or FADH2 (complex II)-linked OXPHOS after Cvs. Another limiting factor of ETC capacity could be the electron flux from glycerol via the glycerophosphate dehydrogenase complex (CGpDH) or from fatty acid ß-oxidation via the electron-transferring flavoprotein complex (ETF) to Coenzyme Q as superordinated factors, as upstream bottleneck of electron flux to complex I and complex II [69]. A similar observation with reduced mitochondrial respiration in regard to complex I, II, and ETC was made following diet-induced vitamin D deficiency in *M. gastrocnemius* of C57BL/6 J mice. Alterations in ETC protein content or citrate synthase activity were excluded and the authors suggested alterations in mitochondrial respiration independent of ETC protein content [70].

The decreased mitochondrial respiration could be explained by decreased basal proton conductance or can also be explained by a change in intrinsic coupling of the respiratory chain activity [71]. It is controversial whether the uncoupling mechanisms of UCP3, the major uncoupling protein expressed in skeletal muscle, affect total EE [72]. Here, there are no differences in UCP3 protein or transcription levels between the Cvs and the control groups. Surprisingly the thermodynamic coupling of mitochondrial phosphorylation is significantly increased in complex II after Cvs, indicating that the rate of energy production is at economic net output power at optimal efficiency. Additionally, the efficiency of both complexes was increased after Cvs. The optimal thermodynamic efficiency (*ƞ-opt*) has been applied to assess biological processes and has been proposed as a measure of mitochondrial function [73]. According to Stucki [74], the values observed suggest that muscle mitochondria adapt their function to maximize ATP production and to maintain cellular phosphate potential at the expense of the energy conversion efficiency after Cvs. The complex II pathway is set toward maximizing the cellular energy state and cellular integrity. In the Cvs group, the complex II mitochondrial set point is associated with a high degree of coupling and high thermodynamic efficiency. The RCR in both complexes was unaltered after Cvs thus does not interfere with a higher degree of mitochondrial coupling. As a result, in vivo mitochondrial ATP synthesis might be more efficient through FADH_2_ (complex II)-linked OXPHOS in Cvs mice. The observation that thermodynamic coupling is increased after Cvs, whereas a correlation of complex II-specific maximal uncoupled respiration with decreased EE, has led to the hypothesis that mitochondria demonstrate functional adaptation with increased efficiency to manage energy balance. This results in a significant increase in economic coupling with the goal of achieving the optimal efficiency of net ATP production rate at the economic benefit to maintain cellular homeostasis processes.

According to our hypothesis, Cvs interferes directly with metabolism but also initiates a memory effect for metabolic adaptation. We show that the direct effects on transcriptome and methylome solely initiated by Cvs are limited in muscle. Here, we specifically looked at the *status quo* after the stress intervention to evaluate any changes present in the transcriptome or even methylome and classify them as persisting after chronic stress. This snapshot of data should provide an indication of molecular changes that are prerequisites for cellular adaptation. Remarkable is the downregulated clock gene Nr1d1 (Rev-erb-α), a transcription factor that is generally involved in energy metabolism featuring maximal oxidative capacity and circadian regulation [75]. Interestingly, most of the significantly repressed transcripts by Cvs are of mitochondrial tRNAs. Specific mitochondrial tRNA mutations have long been linked to type 2 diabetes or metabolic syndrome and diabetes-associated genetic defects [76, 77]. One may speculate that mitochondrial tRNAs are an early target in the decline cascade of mitochondrial function, following stress. Methylome analyses reveal that one of the hypo-methylated genes is the nuclear-encoded mitochondrial tRNA synthase Lars2. Lars2 is expressed in skeletal muscle and controls the translation of mitochondrial-encoded genes via leucyl-tRNA synthetase, the mitochondrial genome stability, and its additional activation improves mitochondrial respiration [78].

In conclusion, our data showed that following Cvs mitochondrial mass is not changed and a correlation occurred of complex II in state 3u with decreased EE. Furthermore, the thermodynamic coupling was increased. This may indicate an early stage of mitochondrial adaptation to Cvs as a compensatory mechanism to manage the energy balance despite advancing atrophy.

### Supplementary Information

Below is the link to the electronic supplementary material.

Supplementary file1 (DOCX 518 KB)

Supplementary file2 (XLSX 1903 KB)

Supplementary file3 (XLSX 37 KB)

Supplementary file4 (XLSX 1791 KB)

## Data Availability

Mass spectrometry data are available from the ProteomeXchange Consortium via the PRIDE partner repository dataset identifier PXD035798. Datasets are available as super series under https://www.ncbi.nlm.nih.gov/geo/query/acc.cgi?acc = GSE210510 or transcriptome dataset accession number GSE210365. Data are available as superseries or methylome data at NCBI GEO https://www.ncbi.nlm.nih.gov/geo/ dataset accession GSE210509).
