# Supplementary material for: Chronic stress targets mitochondrial respiratory efficiency in the skeletal muscle of C57BL/6 mice
Source: Cell Mol Life Sci. 2023 Mar 29;80(4):108. doi: 10.1007/s00018-023-04761-4 (PMC10060325; doi:10.1007/s00018-023-04761-4)
Supplement: Supplementary file 1 — Supplementary file1 (DOCX 518 KB) [file 18_2023_4761_MOESM1_ESM.docx]

Supplementary Material

**Chronic stress targets mitochondrial respiratory efficiency in the skeletal muscle of C57BL/6 mice.**

Aleksandra Nikolic^1,2^, Pia Fahlbusch^1,2^, Natalie Wahlers^1^, Nele-Kathrien Riffelmann^1,2^, Sylvia Jacob^1^, Sonja Hartwig^1,2^, Ulrike Kettel^1,2^, Matthias Dille^1,2,^, Hadi Al-Hasani^1,2,3^, Jörg Kotzka^1,2, #^, Birgit Knebel^1,2,#,^*

^1^Institute of Clinical Biochemistry and Pathobiochemistry, German Diabetes Center at the Heinrich-Heine-University Duesseldorf, Leibniz Center for Diabetes Research; 40225 Duesseldorf, Germany;

^2^German Center for Diabetes Research (DZD), Partner Duesseldorf, 40225 Duesseldorf, Germany

^3^Medical Faculty Heinrich-Heine-University Düsseldorf, 40225 Düsseldorf Germany, 40225 Düsseldorf

^#^both authors contributed equally

***Correspondence:**

Birgit Knebel, PhD

Institute of Clinical Biochemistry and Pathobiochemistry

German Diabetes Center,

Leibniz Center for Diabetes Research

at Heinrich-Heine-University Duesseldorf

Auf‘m Hennekamp 65

40225 Duesseldorf, Germany

E-mail: birgit.knebel@ddz.de

Phone: +49-211-3382 536

## Supplementary Figures

**Supplement figure 1:** **Plasma parameters after 15 days of chronic variable stress intervention.**

Plasma corticosterone, triglyceride (TAG), non-esterified fatty acid (NEFA), and fasted blood glucose levels after chronic variable stress (Cvs). Values are displayed as means ±95 % CI, n = 3-6 animals/ group, single measurements of each animal are shown as dots. Statistics: A Shapiro-Wilk analysis confirmed that the sample data were normally distributed. Plasma analyses were further compared using parametric unpaired t-test, *p<0.05, **p<0.01, ***p<0.001. Ctrl: C57BL/6, Cvs: C57BL/6 after stress intervention.

**
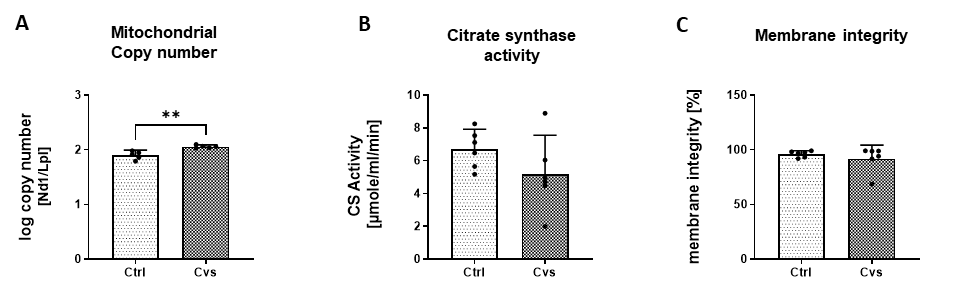
**

**Supplement figure 2:** **Quality control of mitochondrial fractions in regard to content and membrane integrity**

Mitochondrial mass and integrity after Cvs. (**A**) Relative mtDNA/nDNA ratio was analyzed in muscle tissue from Cvs and Ctrl groups (n = 5 animals/ group). (**B**) Citrate synthase activity was measured from fractions of enriched mitochondria (10,000 xg) (n = 6 animals/ per group). (**C**) The percentage of membrane integrity derived from cytochrome-c-oxidase activity of mitochondrial fractions (10,000 xg) from Cvs vs Ctrl muscle tissue (n = 6 animals/ group). Values are displayed as means ±95 % CI. Single measurements of each animal are shown as dots. Statistics: Mann–Whitney test, **p<0.01, Ctrl: C57BL/6, Cvs: C57BL/6 after stress intervention.

**
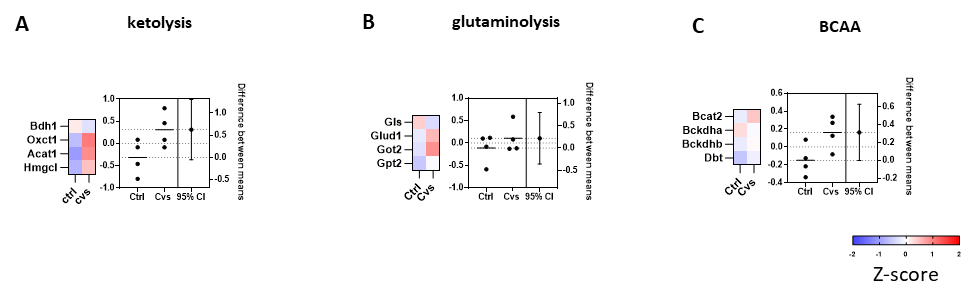
**

**Supplement figure 3:** **Cvs intervention interferes with metabolic pathway component abundance in enriched muscle mitochondria.**

Proteome analyses comparing Ctrl and Cvs group (n = 5 animals/ group). (**A-C**) Z-score plots show the over and under-represented proteins of the indicated pathways. Red identifies upregulation, blue identifies downregulation, and white indicates no change of protein abundance to the mean of each condition with respect to the overall experimental mean. The corresponding estimation plots show on the left axis scatter dot plots with mean z-scores, while dots represent each pathway protein as mean of n=5/ group. On the right axis the mean ±95 % CI alteration in pathway protein abundance of the Cvs and Ctrl comparison is shown. Dotted lines represent the mean of each group centered on 0. (**A**) ketolysis, and (**B**) glutaminolysis, (**C**) Branched chain amino acids (BCAA), Ctrl: C57BL/6, Cvs: C57BL/6 after stress intervention.

**
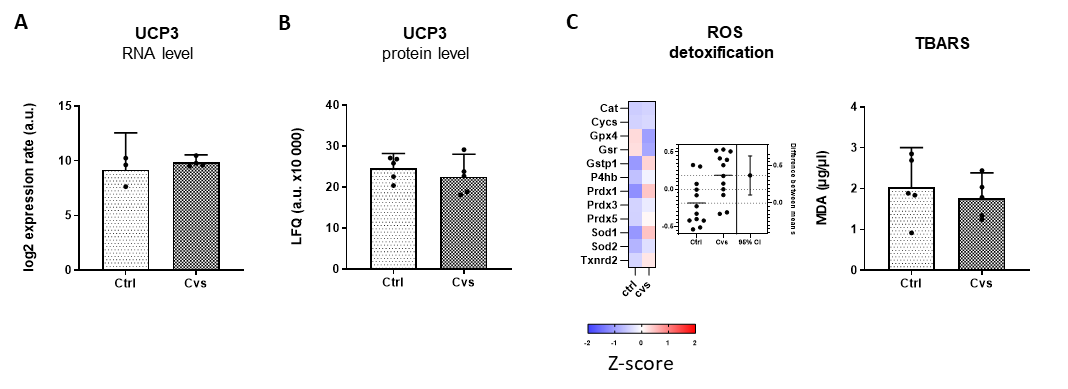
**

**Supplement figure 4:** **Cvs does not affect** **proton leakage in mitochondrial fractions**

(**A**) Log2-fold changes of UCP3 transcript abundance in Cvs and Ctrl muscle (n = 5 animals/ group). (**B**) Label-free quantitation (LFQ) x10 000 of UCP3 protein abundance in Cvs and Ctrl muscle (n = 5 animals/ group). (**C**) Plotted z-score analyses and estimation plot of proteins involved in ROS detoxification. Malondialdehyde (MDA) concentration was measured fluorometrically after reaction with TBARS of 600 xg muscle fraction (n = 5 animals/ group). Data (bar graphs) are expressed as means ±95 % CI. Statistics: Mann–Whitney test, not significant. Single measurements of each animal are shown as dots. The z-score plot shows the over and under-represented proteins of the indicated pathway. Red identifies upregulation, blue identifies downregulation, and white indicates no change of protein abundance to the mean of each condition with respect to the overall experimental mean. The corresponding estimation plot shows on the left axis scatter dot plots with mean z-scores, while dots represent each pathway protein as mean of n = 5/ group. On the right axis the mean ±95 % CI alteration in pathway protein abundance of the Cvs and Ctrl comparison is shown. Dotted lines represent the mean of each group centered on 0. Ctrl: C57BL/6, Cvs: C57BL/6 after stress intervention. TBARS: Thiobarbituric Acid Reactive Substances; ROS: reactive oxygen species.

**
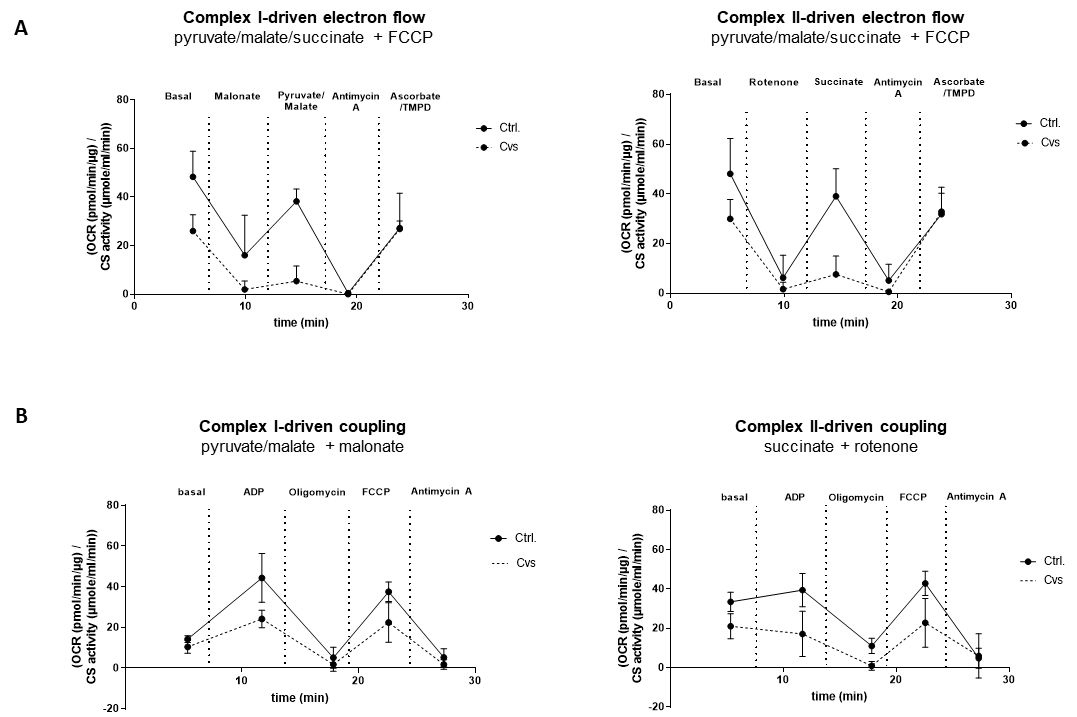
**

**Supplement figure 5: Electron flow capacity and coupling efficiency of mitochondrial ETC after Cvs, injection time-line diagrams**

Electron flow capacity and coupling efficiency of mitochondrial ETC after Cvs. Respiratory capacity was measured in the enriched mitochondrial fractions of isolated muscle mitochondria from Cvs compared to Ctrl muscles in response to ETC manipulation. The dotted vertical lines refer to the time point at which the listed compound was injected. Shown is the oxygen consumption rate (OCR) recorded during the time course of the experiment. (**A**) Electron transport was measured in the uncoupled state (induced by FCCP) specific for complex I (left) and complex II (right) –driven electron transport. OCR at basal level was measured in the presence of unlimited substrate condition (pyruvate/malate/succinate), then OCR was measured after serial injection of complex-specific inhibitor malonate or rotenone, complex-specific substrate stimulation by injection pyruvate/ malate or succinate, this was followed by Antimycin A and Ascorbate/TMPD injections. (**B**) Coupling experiments were performed by measuring OCR individually for complex I (left) and complex II (right). Complex I-specific OCR was measured using complex I-specific substrate pyruvate and malate, with inhibition of complex II activity by malonate. Complex II-specific OCR was measured using complex II-specific substrate succinate with inhibition of complex I activity by rotenone. Mitochondrial respiration was measured at basal experimental condition and in response to serial injection of ADP, oligomycin, FCCP and Antimycin A. Data points represent the mean of n = 6 animals/ group shown as mean ±95 % CI. All data evaluation and interpretations are given in Figure 5, main document. Ctrl: C57BL/6, Cvs: C57BL/6 after stress intervention.

**
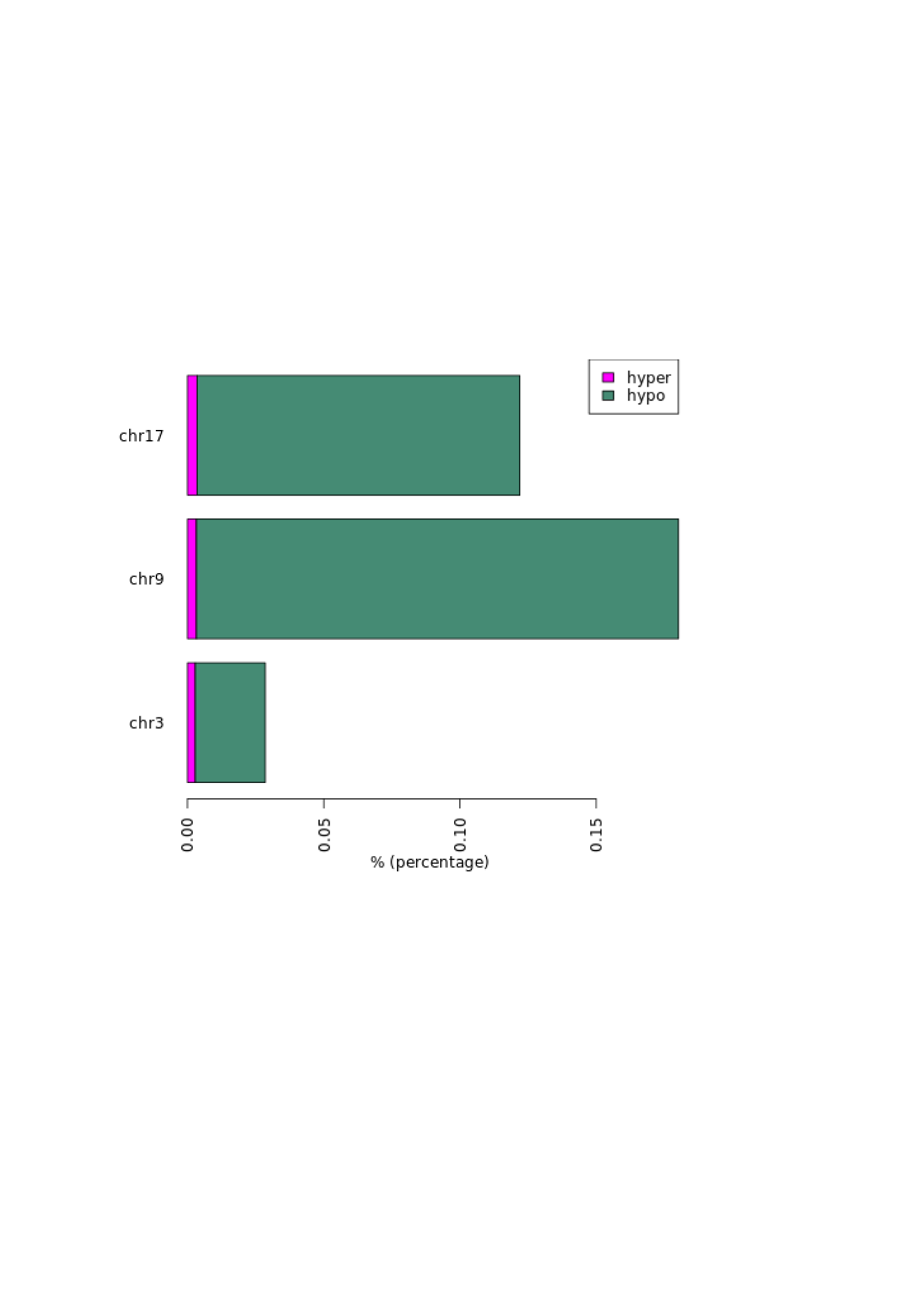
**

**Supplement figure 6: Differential methylated areas after Cvs intervention in *gastrocnemius muscle*.**

Hyper and hypomethylated regions per chromosome as identified in differential methylation analyses (q-value <0.01, methylation difference >2 %; MethylKit, (BaseSpaceLabs, Illumina San Diego, USA); (n = 5 animals/ group)). Corresponding data are given in Supplement Table 2, Datasheet “Differential Methylation”.





**Supplement Figure 7: Cvs does not affect** **DNA modifying enzyme activity**

Enzyme activity of methyltransferases (MTase) and sirtuins (histone deacetylases class III: SIRT1 to SIRT7), in muscle from Cvs compared to Ctrl mice (n = 6 animals/ group). Data are presented as mean ±95 % CI. Single measurements of each animal are shown as dots. Statistics: Mann–Whitney test, not significant. Ctrl: C57BL/6, Cvs: C57BL/6 after stress intervention.

**
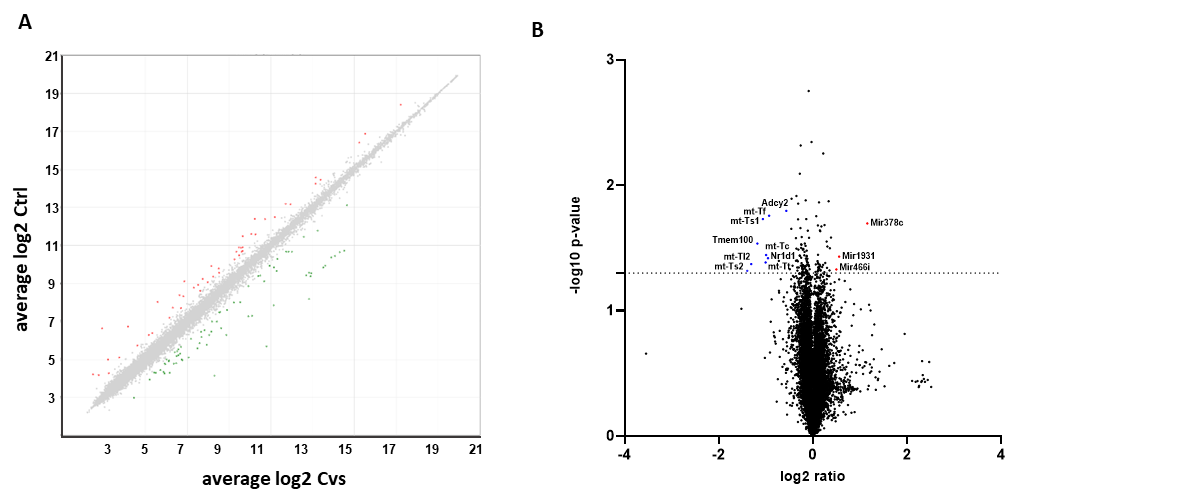
**

**Supplement Figure 8**: **Transcriptome analyses of *gastrocnemius muscle* after Cvs.**

(**A**) Scatter plot with 1.2-fold difference (y-axis: Avg Log2 condition Ctrl; x-axis Avg Log2 condition CVS). (**B**) The log2 fold changes of transcriptome data abundance in Cvs compared to Ctrl mice (n = 5 animals/ group). 14084 RNA transcripts were detected. Upregulated (Red; n = 3) or downregulated (blue; n = 10) were determined by Student’s t-test (p<0.05) with 1.2- fold regulation. Corresponding data are given in Supplement Table 3.
